# Brain plasticity underlying acquisition of new organizational skills in children: A Rashomon analysis

**DOI:** 10.3389/fnimg.2025.1671310

**Published:** 2025-12-09

**Authors:** Luis Martinez Agulleiro, Bowen Deng, Richard Gallagher, Howard B. Abikoff, Yuliya Yoncheva, Lauren Robinson, Greta Conlon, Maleeha Haroon, Chao-Gan Yan, Adriana Di Martino, Yihong Zhao, F. Xavier Castellanos

**Affiliations:** 1 Department of Child and Adolescent Psychiatry, New York University Grossman School of Medicine, New York, NY, United States; 2 Deparment of Psychological and Cognitive Sciences, Tsinghua University, Beijing, China; 3 Autism Center, Child Mind Institute, New York, NY, United States; 4 Columbia University School of Nursing, Columbia University, New York, NY, United States

**Keywords:** organizational skills, attention-deficit/hyperactivity disorder, resting state, neuroimaging, executive functions

## Abstract

**Objective:**

We used resting-state functional magnetic resonance imaging to identify changes in brain functional connectivity (FC) associated with Organizational Skills Training (OST).

**Method:**

In an open, waitlist-controlled, randomized clinical trial (NCT04108273), 51 children aged 8–12 years with deficient organizational skills were assigned to immediate tele-health OST treatment (twice weekly, 10 weeks) or waitlist. We obtained Children’s Organizational Skills Scale-Parent version (COSS-P) scores and examined FC changes between dorsal anterior cingulate cortex (dACC) and preregistered subcortical anterior ventral striatum (aVS) regions-of-interest.

**Results:**

OST produced significantly lower COSS-P scores compared to waitlist, with a large effect size (Cohen’s f^2^ = 0.77). Initial imaging analyses revealed a significant increase (instead of the predicted decrease) in FC between dACC and the aVS component of the default mode network in the immediate treatment group (ΔFC = 0.092 ± 0.041, 95% CI [0.009, 0.175], *p* < 0.05). Analyses were then performed with two additional analytic pipelines, neither of which detected any significant effects.

**Conclusion:**

Although improvements in organizational deficits were associated with increased FC within a circuit linking dACC and the default mode network region of the aVS in one analysis, the direction was the opposite of predicted and results did not replicate. Thus, we highlight the tentativeness of our findings; we have de-identified all the data and made it available for investigators to examine and to combine with other datasets in mega- and meta-analyses. Future studies should also include alternative control conditions and larger samples.

**Clinical trial registration:**

https://clinicaltrials.gov/study/NCT04108273?cond=NCT04108273&rank=1.

## 1 Introduction

Impairment in organizational skills is frequently observed in patients with attention-deficit/hyperactivity disorder (ADHD), but is also prevalent across other neurodevelopmental conditions, such as autism spectrum disorder or specific learning disorders (Li et al., 2024). Such deficits substantially impair academic performance, occupational attainment and social functioning, often persisting into adulthood and contribute to significant long-term challenges (Barkley and Fischer, 2011). While psychostimulants improve attention and hyperactivity symptoms in patients with ADHD (Catalá-López et al., 2017; Cortese, 2020) they do not ameliorate organizational skills deficits (Abikoff et al., 2009).

This gap has led to the development of several efficacious behavioral interventions focused on executive function rehabilitation for children and adolescents with ADHD, such as the Group Based Intervention of Deficits in Attention and Executive Functions (Paananen et al., 2022), the Collaborative Life Skills Program (Pfiffner et al., 2013), and Central Executive Training (Chan et al., 2023). Organizational Skills Training (OST), an intervention devised by Abikoff et al. (2013) yielded robust effects for both parent- and teacher-reported measures of organizational skills, with large effect sizes (Hedges’ g = 2.00, 95% CI [1.50, 2.50]; and g = 1.04, 95% CI [0.59, 1.48]), respectively (Bikic et al., 2017). Importantly, improvements in a two-site randomized controlled trial were sustained 14 months later (Abikoff et al., 2013).

Evidence substantiating the enduring efficacy of OST in eliciting robust behavioral improvements suggests changes in brain functional circuits (Kelly and Castellanos, 2014). This warrants examination to identify potential therapeutic targets. In this regard, resting-state functional magnetic resonance imaging (R-fMRI) is a suitable method for delineating changes in macroscale brain circuitry, especially in children for whom task-based approaches may be impractical (Kelly et al., 2012).

Previous R-fMRI studies have implicated the dorsal anterior cingulate cortex (dACC) in the regulation of cognitive control processes (Weissman et al., 2006; Sheth et al., 2012; Simões-Franklin et al., 2010; Castellanos et al., 2008). In a small pilot sample (*n* = 15), we observed that intrinsic functional connectivity (iFC) between dACC and a subregion of the anterior ventral striatum (aVS) was decreased compared to baseline scans in children with ADHD and deficient organizational skills after OST (Chen et al., 2016). Notably, functional connectivity analysis of the striatum has revealed three distinct subcortical iFC-defined regions corresponding to the default mode (DMN), frontoparietal (FP) and limbic (LIM) networks (Choi et al., 2012).

Building on these insights, we conducted a randomized clinical trial to (1) validate the efficacy of OST in improving organizational skills deficits in a trans-diagnostic sample of school-aged children; and (2) determine whether OST induces changes in brain functional connectivity, specifically by modifying iFC between dACC and specific subcortical functional networks within the aVS (i.e., aVS-DMN, aVS-FP and/or aVS-LIM). We hypothesized that OST would improve COSS-P scores and decrease the iFC between dACC and one or more of our three pre-registered aVS masks.^1^

## 2 Methods

### 2.1 Study design and participants

**Figure 1:**
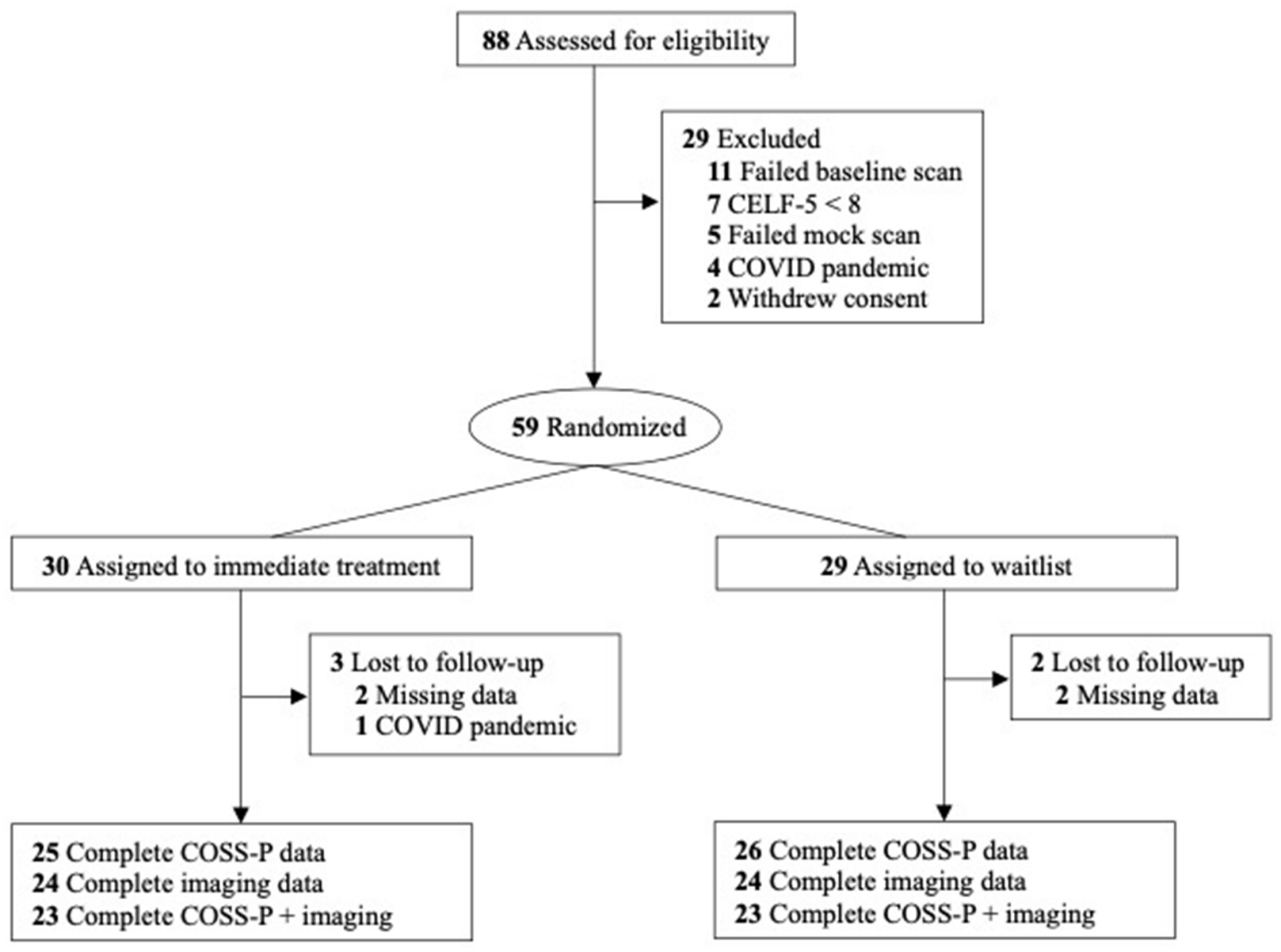
CONSORT diagram of participant flow.

The study protocol was registered in ClinicalTrials.gov (NCT04108273). Participants of this open, waitlist-controlled, randomized clinical trial underwent R-fMRI scans twice: once at baseline and again within 2 weeks postintervention (or 10 to 12 weeks after baseline for the control group; the intervention lasted 10 weeks). Block randomization was performed, stratifying for sex assigned at birth and stimulant treatment status. To address the historical under-recruitment of females in ADHD studies (Pereira-Sanchez et al., 2021), we prioritized enrollment of female participants, with a goal of obtaining at least 30% females. Details on sample recruitment and randomization can be found in Figure 1. Parents/legal guardians of all participants provided written informed consent and participants provided written assent. All study procedures were approved by the NYU Langone Health Institutional Review Board. The study followed the Consolidated Standards of Reporting Trials (Schulz et al., 2010) and the Best Practices in Data Analysis and Sharing in Neuroimaging using MRI (Nichols et al., 2017).

Participants were recruited from October 2019 to February 2023. We included children with deficient organizational skills (defined as having a Children’s Organizational Skills Scale-Parent version [COSS-P] Total score ≥1 SD [T-score ≥60] above age and sex-based norms, along with confirmation of associated impairment on COSS impairment items), regardless of diagnosis. Participants were 8–12 years of age and attended elementary school grades 3–5, as these are the grades for which the intervention was designed (Abikoff et al., 2013; Gallagher et al., 2014). Additionally, participants were required to have estimated IQ ≥ 85 on the Wechsler Abbreviated Scale of Intelligence, 2nd Edition (Wechsler and Zhou, 2011), and Formulated Sentences and Understanding Spoken Paragraphs scores ≥8 on the Clinical Evaluation of Language Fundamentals, 5th Edition (Wiig et al., 2013). These criteria assured that children were able to follow the instructions and demands from this highly verbal treatment program. No restriction on handedness preference was imposed. For participants receiving psychostimulant medication, a stable dose for at least 1 month before study entry was required.

Although this transdiagnostic study did not require participants to meet criteria for a specific diagnosis, parents were queried with the computerized version of the Kiddie Schedule for Affective Disorders and Schizophrenia (Townsend et al., 2020), followed by a confirmatory clinical interview performed by a clinical psychologist (MH).

Participants were excluded if they were unable to provide baseline imaging data (including failure to remain sufficiently still during mock-scan training), had recent (past 6 months) or current history of neuroleptic treatment or current treatment with other psychotropic medications (except psychostimulants); history of medical illness requiring chronic treatment, intrathecal chemotherapy or focal cranial irradiation; premature birth (<32 weeks estimated gestational age) or birth weight <1,500 g; history of leukomalacia or static encephalopathy; intracerebral hemorrhage beyond grade 2; other specific or focal neurological or metabolic disorders (including epilepsy, except for resolved febrile seizures); history of traumatic brain injury; or contraindication for MRI scanning (e.g., metal implants, pacemakers or metal foreign bodies). The mandatory COVID-19 shutdown prevented the second MRI assessment of five participants who were thus excluded from analyses.

### 2.2 Intervention

OST is a targeted behavioral intervention designed to improve organizational skill deficits, focusing on domains known to impact academic performance adversely, i.e., tracking assignments, managing materials and time management and planning (Abikoff et al., 2009; Gallagher et al., 2014) The intervention encompasses multifaceted components, including parent training, teaching the children new tools and routines to improve their organization skills, and active engagement of teachers. Based on parent feedback regarding the burden of participating in 20 twice weekly one-hour in-person sessions, we adapted OST to ten 1-hour virtual weekly sessions (involving both the participant and parent) and ten 30-min virtual booster sessions 2–3 days later (focusing on parent guidance and child demonstration of skills taught in the longer, prior session). These shorter sessions were designed to help parents utilize praise and rewards for skill use as effectively as possible and another opportunity for children to engage in therapist-guided practice of the skills. Teachers were informed of the skills taught via scheduled phone calls or secure emails and they provided feedback to parents using a daily written checklist.

### 2.3 Measures

Organizational skills were assessed using the Children’s Organizational Skills Scale–Parent version (COSS-P), a comprehensive and validated measure of organizational skills for children aged 8–13 years (Abikoff and Gallagher, 2009). The COSS-P is a parent-reported questionnaire that assesses a child’s overall competence in managing task demands at home and school and delineates competence in planning, tracking assignments, and managing materials and time. Higher COSS-P scores represent worse (i.e., more deficient) organizational skills. This scale was established based on more than 5,000 assessments of children, including normative sampling stratified to the US general population. COSS-P scores were obtained at baseline (before the intervention and within 2 weeks of the baseline scan) and within 2 weeks after the end of the intervention (or 10–12 weeks after baseline for the control group; the intervention lasted for 10 weeks). The primary endpoint of the present study was change in COSS-P Total score. Secondary endpoints included changes in each of the three COSS-P subscales (i.e., Task Planning, Organized Actions, and Memory and Materials Management). Although we had planned to also obtain teacher versions of the COSS, the institutional review board of the local public school system declined our request to have teachers fill out the forms, because participation in research was deemed not an educationally essential function.

### 2.4 Image acquisition and preprocessing

Within a week prior to the MRI session, children were familiarized with the scanner environment and practiced keeping still in a mock scanner to reduce motion artifacts during scanning. MRI data were acquired with a Siemens 3-Tesla Prisma scanner with XR Gradients and a 32-channel Siemens head coil. The resting-state sequences were acquired at the beginning of each scanning session, with participants asked to hold still and keep their eyes open while a centrally located black cross was presented against a white background. Foam padding and headphones were used to limit head motion and reduce scanner noise.

Resting-state functional images were collected using T2*-weighted echo planar (EPI) pulse sequence imaging depicting the blood oxygen-level dependent signal (TE = 30 ms, TR = 800 ms, flip angle = 55°, spatial resolution 2.4 × 2.4 × 2.4 mm isotropic voxels, transverse orientation, slice thickness = 2.4 mm, 66 slices fully covering the cerebral cortex and the cerebellum, 525 volumes, acquisition time = 7 min). For anatomical reference a high-resolution isotropic magnetization prepared rapid gradient echo with prospective motion correction sequence, consistent with the Adolescent Brain Cognitive Development study neuroimaging protocol (Casey et al., 2018), was acquired following the EPI sequences (TE = 2.24 ms, TR = 2,400 ms, flip angle = 8°, sagittal orientation, spatial resolution 0.8 × 0.8 × 0.8 mm isotropic voxels, slice thickness = 0.8 mm).

Due to conflicting findings (see Results), data preprocessing and analysis was performed separately with three different software suites: Data Processing Assistant for Resting-State fMRI (DPARSF), Configurable Pipeline for the Analysis of Connectomes (C-PAC) and the CONN toolbox.

#### 2.4.1 Data processing assistant for resting-state fMRI (DPARSF)

The Data Processing Assistant for Resting-State fMRI (DPARSF)^2^ is based on Statistical Parametric Mapping (SPM)^3^ and the toolbox for Data Processing & Analysis of Brain Imaging (Yan and Zang, 2010; Yan et al., 2016). With DPARSF, the first 4 time-points of each functional run were discarded to allow magnetization to reach steady state. Image preprocessing included slice time correction, motion correction (using the Friston-24 head motion parameters), nuisance regression (white matter, cerebrospinal fluid) with and without global signal regression (GSR), segmentation-based normalization using the diffeomorphic anatomical registration through exponentiated Lie algebra tool and non-linear registration of functional images to Montreal Neurological Institute space (version MNI152NLin2009). The normalized functional images were then spatially smoothed with a Gaussian kernel with a full width at half maximum of 4 mm, the DPABI default. Finally, bandpass temporal filtering (0.01–0.1 Hz) was applied to the smoothed images to reduce the effect of low-frequency drift and high-frequency physiological noise. Here we report results with global signal regression (GSR; analyses without GSR yielded similar results as shown in Supplementary material).

#### 2.4.2 Configurable pipeline for the analysis of connectomes (C-PAC)

The Configurable Pipeline for the Analysis of Connectomes (C-PAC)^4^ is an open-source software platform designed for the automated preprocessing and analysis of resting-state fMRI data, integrating several established neuroimaging tools (Craddock et al., 2013). We employed the default C-PAC pipeline, adjusted to match the preprocessing parameters used in DPARSF wherever possible.

Briefly, anatomical processing included non-linear transformation between the skull-on T1-weighted anatomical image and the MNI152 2 mm brain-only template, skull-stripping using a combination of intensity-based and template-based methods and creation of tissue segmentation maps in MNI space at 2 mm resolution. Functional preprocessing included slice timing correction and motion correction using a two-stage approach in which the images were first coregistered to the mean fMRI and then a new mean was calculated and used as the target for a second coregistration.

Nuisance variable regression (NVR) was performed on motion corrected data using a second-order polynomial, a 24-regressor model of motion, 5 nuisance signals (identified via principal components analysis of signals obtained from white matter), and the mean cerebrospinal fluid signal. The NVR procedure was performed twice, with and without GSR as a nuisance regressor (analyses without GSR yielded similar results as shown in Supplementary material). The NVR residuals were bandpass filtered (0.01–0.1 Hz), written into MNI space at 3 mm resolution and subsequently smoothed using a 4 mm full width at half maximum kernel. The pipeline configuration file is available in.^5^

#### 2.4.3 CONN toolbox

Analyses of the same fMRI data were also performed using CONN (RRID: SCR_009550) release 22.v2407 (Nieto-Castanon and Whitfield-Gabrieli, 2022) and SPM (RRID: SCR_007037) release 12.7771 (Penny et al., 2011). Briefly, functional and anatomical data were preprocessed using a modular preprocessing pipeline including realignment with correction of susceptibility distortion interactions, slice timing correction, outlier detection, direct segmentation, MNI-space normalization and smoothing. In addition, we applied a standard denoising pipeline including the regression of potential confounding effects, followed by bandpass frequency filtering (0.01–0.1 Hz). A detailed description of the CONN preprocessing procedure can be found in Supplementary material.

### 2.5 Statistical analysis

Data analyses, including group level imaging analyses (except CONN), were performed using R (version 4.2.2), with statistical significance set at *p* < 0.05, uncorrected. A descriptive analysis was used to summarize the data. Categorical variables between groups were compared using χ^2^ tests, while *t*-tests were used for between-group comparisons of continuous variables.

To assess the efficacy of OST we performed a linear regression analysis with the change of COSS-P scores (i.e., post–pre; ΔCOSS-P) as the dependent variable and randomization group (immediate treatment or waitlist) as the independent variable, controlling for age and sex. For this analysis, we included all participants with complete COSS-P data (*N* = 51: immediate treatment *n* = 25, waitlist *n* = 26), regardless of available imaging data.

To test whether OST decreased iFC between dACC and the aVS functional networks, we performed a linear regression analysis with iFC change (ΔiFC) between dACC and each aVS cluster (aVS-DMN, aVS-FP and aVS-LIM) (post–pre) as the dependent variable and randomization group as the independent variable, controlling for age and sex. The dACC seed was defined as a 7.5-mm diameter sphere centered at MNI coordinates (Chan et al., 2023; Pfiffner et al., 2013; Liljeholm and O'Doherty, 2012), as reported previously (Weissman et al., 2006; Castellanos et al., 2008). The three aVS ROIs spanned three distinct iFC-defined clusters corresponding to DMN, FP and LIM (Choi et al., 2012). These clusters were preregistered and are publicly available in the Open Science Framework repository.^6^ The average time-series of all voxels in each cluster/seed was extracted and time-series correlation analyses were performed to calculate the iFC between dACC and each aVS ROI. These analyses were restricted to participants with complete COSS-P and imaging data (*N* = 46: immediate treatment *n* = 23, waitlist *n* = 23).

For CONN, individual- and session-level connectivity matrices between dACC and each aVS subnetwork were estimated, characterizing the iFC with Fisher-transformed bivariate correlation coefficients from a weighted general linear model. Group-level analyses were performed using a general linear model within CONN that replicated the previously described procedure with R, restricted to the same subjects.

## 3 Results

### 3.1 Effectiveness of OST on organizational skills deficits

Table 1 summarizes the demographic and clinical characteristics of the sample and their effective randomization. Overall, 15 participants (32.6%) had a diagnosis of only ADHD, while 24 (52.2%) exhibited a range of comorbid conditions as shown in Table 1. Five participants (10.9%) did not meet full criteria for ADHD but were diagnosed with other conditions: adjustment disorder (*n* = 1), autism-spectrum disorder (*n* = 2), specific learning disorder (*n* = 1), motor disorder (*n* = 1) and oppositional defiant disorder (*n* = 1). Two participants (4.3%) did not meet full criteria for any current psychiatric diagnosis at enrollment. Head motion during R-fMRI was minimal (mean framewise displacement [SD] = 0.10 [0.02]). Participant-specific diagnoses and other phenotypic deidentified data are provided at.^7^

**Table 1:** Demographic and clinical characteristics of the participants with complete COSS-P and imaging data at baseline.

Characteristic	Overall (*N* = 46)	Treatment (*n* = 23)	Waitlist (*n* = 23)	*p*-value
Age, mean (SD)	9.3 (0.9)	9.4 (1.0)	9.4 (0.7)	1.0
Sex assigned at birth, *n* (%)				1.0
Male	26 (56.5)	13 (56.5)	13 (56.5)	
Female	20 (43.5)	10 (43.5)	10 (43.5)	
Race, *n* (%)				0.14
Asian	3 (6.5)	–	3 (13.0)	
Black	1 (2.2)	–	1 (4.3)	
Multiracial	10 (21.7)	4 (17.4)	6 (26.1)	
White	30 (65.2)	17 (73.9)	13 (56.5)	
Not available	2 (4.3)	2 (8.7)	–	
Ethnicity, *n* (%)				0.19
Hispanic/Latino	6 (13.0)	5 (21.7)	1 (4.3)	
Not Hispanic/Latino	40 (87.0)	18 (78.3)	22 (95.7)	
ADHD, *n* (%)	39 (84.8)	18 (78.3)	21 (91.3)	0.41
Adjustment disorder, *n* (%)	2 (4.3)	1 (4.3)	1 (4.3)	1.0
Anxiety disorder, *n* (%)	10 (21.7)	2 (8.7)	8 (34.8)	0.07
Autism-spectrum disorder, *n* (%)	3 (6.5)	2 (8.7)	1 (4.3)	1.0
Learning disorder, *n* (%)	6 (13.0)	3 (13.0)	3 (13.0)	1.0
Motor disorder, *n* (%)	6 (13.0)	1 (4.3)	5 (21.7)	0.19
Oppositional defiant disorder, *n* (%)	9 (19.6)	5 (21.7)	4 (17.4)	1.0
Estimated IQ, mean (SD)	117 (12.4)	118 (13.0)	116 (11.9)	0.56
CELF-5-FS, mean (SD)	12.2 (2.0)	11.9 (2.1)	12.6 (1.8)	0.21
CELF-5-USP, mean (SD)	10.8 (2.0)	10.8 (2.3)	10.8 (1.6)	1.0
mFD, mean (SD)	0.10 (0.02)	0.10 (0.02)	0.09 (0.03)	0.60
mFD ≥ 0.1 mm, *n* (%)	15 (32.6)	9 (39.1)	6 (26.1)	0.53
dACC-aVS iFC, mean (SD)				
Default mode network	0.22 (0.17)	0.26 (0.17)	0.17 (0.16)	0.09
Frontoparietal network	0.29 (0.15)	0.31 (0.17)	0.28 (0.13)	0.49
Limbic network	0.16 (0.17)	0.18 (0.17)	0.15 (0.17)	0.54
COSS-P T-score, mean (SD)				
Total	69.2 (6.8)	68.5 (6.0)	69.9 (7.6)	0.49
Task planning	64.4 (9.3)	62.2 (9.8)	66.7 (8.4)	0.11
Organizes actions	62.8 (4.0)	62.0 (3.5)	63.7 (4.4)	0.14
Memory and materials management	66.3 (9.1)	67.0 (8.9)	65.6 (9.5)	0.61

ADHD, attention-deficit/hyperactivity disorder; aVS, anterior ventral striatum; CELF-5, Clinical Evaluation of Language Fundamentals, 5th Edition; COSS-P, Children’s Organizational Skills Scale parent-version; dACC, dorsal anterior cingulate cortex; FS, Formulated Sentences subscale; iFC, intrinsic functional connectivity; IQ, intelligence quotient; mFD, mean framewise displacement; USP, Understanding Spoken Paragraphs subscale.

As shown in Table 2, for participants with complete COSS-P data (N = 51), after controlling for age and sex assigned at birth, we found a significantly greater reduction (*p* < 0.0001) of COSS-P Total scores in the immediate treatment group relative to the waitlist group. The intervention accounted for 45% of the variance in the change of COSS-P Total scores, with a large effect size (Cohen’s *f*^2^ = 0.77). Secondary analysis revealed statistically significant reductions in all COSS-P subscales in the immediate treatment group compared to waitlist, although less robustly for Task Planning than for Memory and Materials Management and for Organized Actions (Table 2).

**Table 2:** Change in COSS-P scores after the intervention and waitlist period.

ΔCOSS-P T-score, mean (SD); [95% CI]	Immediate treatment (*n* = 25)	Waitlist (*n* = 26)	*p*-value	Explained variance (%); effect size (Cohen’s *f*^2^)
Total	−14.7 (1.5); [−17.7, −11.8]	−2.6 (1.5); [−5.6, 0.3]	<0.0001	45.4; 0.77
Task planning	−12.2 (2.0); [−16.3, −8.1]	−4.9 (2.0); [−9.0, −0.9]	0.014	11.7; 0.13
Organizes actions	−9.6 (1.2), [−12.0, −7.2]	−2.1 (1.2); [−4.5, 0.2]	<0.0001	31.4; 0.45
Memory and materials management	−13.9 (1.8); [−17.4, −10.3]	−1.5 (1.8); [−5.1, 2.0]	<0.0001	<0.0001

COSS-P, Children’s organizational skills scale parent-version.

Notably, COSS-P scores of the immediate treatment group reached the normative range (i.e., T-score < 60) after the intervention (mean [SD]: COSS-P Total 54.1 [7.0]; COSS-P Task Planning 50.8 [6.3]; COSS-P Organized Actions 52.8 [8.1]; and COSS-P Memory and Materials Management 53.0 [6.5]) while the waitlist group scores remained at least one standard deviation above the normative mean (mean [SD]: COSS-P Total 65.8 [7.6]; COSS-P Task Planning 60.7 [9.7]; COSS-P Organized Actions 60.1 [6.3]; and COSS-P Memory and Materials Management 62.6 [9.5]).

As the prior OST trial required a diagnosis of ADHD, we wondered if the effect of treatment differed by ADHD diagnostic status. Group mean comparisons did not yield statistically significant differences in response to treatment between ADHD (*n* = 43) and non-ADHD (*n* = 8) participants (mean [SD] ΔCOSS-P Total score in non-ADHD participants: −11.3 [7.7] vs. ADHD participants: −8.18 [9.4], *p* = 0.414; ADHD status * randomization group interaction term: *β* = 2.96, *p* = 0.640).

### 3.2 Effect of OST on brain connectivity

#### 3.2.1 Results from DPARSF

After controlling for age and sex, for children with both complete COSS-P data and imaging data (*N* = 46), linear regression models yielded a statistically significant effect of treatment group in ΔiFC between dACC and aVS-DMN (*β* = 0.120, 95% CI [0.004, 0.237], *p* = 0.04). The intervention explained 7.4% of the variance in ΔiFC between dACC and aVS-DMN (Cohen’s *f*^2^ = 0.07). The immediate treatment group showed a statistically significant increase in iFC between dACC and aVS-DMN (mean [SD] ΔiFC = 0.092 [0.041], 95% CI [0.009, 0.175]), while the waitlist group showed a non-significant decrease in iFC between dACC and aVS-DMN (mean [SD] ΔiFC = −0.029 [0.041], 95% CI [−0.111, 0.054]). No statistically significant group differences were found in ΔiFC between dACC and aVS-FP (*β* = 0.072, 95% CI [−0.039, 0.184], *p* = 0.196) or between dACC and aVS-LIM (*β* = 0.028, 95% CI [−0.077, 0.132], *p* = 0.596).

#### 3.2.2 Results from C-PAC and CONN

Our initial results, although statistically significant, were in the opposite direction from what we had found in our pilot study (Chen et al., 2016) and hypothesized. Seeking to confirm the reliability of this unexpected result, we reanalyzed using two additional software suites.

Findings from C-PAC yielded no statistically significant group differences in ΔiFC between dACC and the aVS ROIs: aVS-DMN (β = 0.02, 95% CI [−0.075, 0.134], *p* = 0.576), dACC and aVS-FP (*β* = 0.006, 95% CI [−0.106, 0.117], *p* = 0.920) and dACC and aVS-LIM (*β* = −0.053, 95% CI [−0.156, 0.051], *p* = 0.311).

Similarly, analyses performed with CONN did not identify statistically significant changes in ΔiFC between dACC and aVS-DMN (*β* = −0.001, *p* = 0.988), dACC and aVS-FP (*β* = 0.04, *p* = 0.520) or dACC and aVS-LIM (*β* = −0.01, *p* = 0.907).

## 4 Discussion

The present registered (NCT04108273^8^) waitlist-controlled, randomized clinical trial further confirms the efficacy of OST for improving organizational skills deficits among elementary school-aged children with neurodevelopmental conditions, even when administered virtually. This was evidenced by substantial reductions in COSS-P scores with a large effect size. Results provide support for the utility of a telehealth version of OST, extending its potential availability. In keeping with the transdiagnostic nature of organizational skills deficits, we included children regardless of diagnosis, supporting the generalizability of OST beyond ADHD.

Nevertheless, our analyses of the impact of OST on brain connectivity were inconclusive. Our initial results yielded a significant increase in iFC between dACC and our DMN aVS ROI. On one hand, prior research has indicated that reduced structural connectivity between these regions in children with ADHD correlates with increased delay discounting, a measure that reflects poor planning and deficient organizational skills (Rosch et al., 2023). On the other hand, increased resting-state iFC between these regions has been linked to improved self-control in individuals with internet gaming disorder (Gong et al., 2022). Structural and functional alterations of this circuit have also been detected in conditions characterized by disrupted goal-directed behaviors, such as apathetic syndrome (Le Heron et al., 2018). This circuit is involved in processing motivationally relevant information (Onoda et al., 2008; Calabrese et al., 2022; Liljeholm and O'Doherty, 2012), planning goal-directed behaviors (Haber and Knutson, 2010) and evaluating the cost–benefit of planned and controlled actions (Le Heron et al., 2018; Magno et al., 2006; McGovern and Sheth, 2017). These findings situate the circuit connecting dACC and VS as a central component of organizational skills and executive function, particularly in monitoring ongoing actions and detecting unexpected errors (Cardinal et al., 2002), thus allowing individuals to learn which behaviors will be reinforced in the future (Le Heron et al., 2018).

However, our results were in the opposite direction to what we had expected. Given the sensitivity of imaging results to analytical pipelines (Botvinik-Nezer et al., 2020), we sought to replicate the results on alternative pipelines. However, neither alternative method yielded any credible significant differences. This variability underscores the inherent difficulty of establishing highly reliable results in neuroimaging research. Given the rapid rate of development of neuroimaging analytical methods and models, we withhold drawing definitive conclusions from this “*Rashomon* (Kurosawa, 1951) analysis.” We recognize the risk that investigators may, consciously or not, favor results that align with their hypothesis or interpretive preferences; rather, we wish to communicate the importance of reporting results with full transparency and resisting the temptation to apply post-hoc explanations for unexpected albeit statistically significant results. Further, we have made our data openly available to facilitate future aggregation with other datasets and reanalysis as methodological and processing approaches advance. Ultimately, only through convergence across independent studies and analytical strategies can the field approach a more reliable understanding of the underlying phenomena of brain function.

Additionally, our study provides a critical foundation for future research. By openly sharing this high-quality (low head-motion) dataset, we aim to facilitate further analyses, mega-analyses, and educational and training initiatives for emerging researchers. The specific effects of ADHD medications on brain functional connectivity are unclear, although they have been hypothesized to influence cortico-striato-thalamo-cortical circuits and connectivity between DMN and the FP network with other cortical and subcortical regions (Pereira-Sanchez et al., 2021). Here, in one of three analyses, we found that OST increased the iFC between dACC and the striatal component of the DMN, aVS-DMN.

Despite our inconclusive findings, our study possesses several notable strengths, including its pre-registered randomized clinical design with registered ROIs reflecting an explicit hypothesized target. Rather than taking the striatum as a single unit, we used a functional parcellation that captures its functional heterogeneity, allowing us to begin to parse its complex functional roles. Future studies should apply these or newer striatal parcellations (Seitzman et al., 2020; Zhang et al., 2010; Greene et al., 2014; Bell and Shine, 2016). Our within-subject design mitigated the effects of individual differences, increasing statistical power from our moderately-sized sample. Additionally, the high-quality of our data, characterized by minimal head motion, reduced the likelihood of type 1 errors. To make such laboriously collected data broadly available, they can be openly accessed in,^9^ including fMRI sequences not examined in this manuscript (e.g., *Inscapes* (Vanderwal et al., 2015) and a short narrative film).

Nevertheless, limitations were also notable. Planned sample size (*N* = 68 for the randomly controlled trial) was markedly reduced by the COVID-19 pandemic. We were unable to obtain COSS ratings from local public-school teachers, as their Institutional Review Board determined that assisting in research falls outside of their educational functions. Thus, we relied on parent ratings exclusively. The use of a waitlist control group limits our ability to definitively link our findings to OST per se. Implementing attention control groups (i.e., an alternative intervention) (Abikoff et al., 2009) in future studies will be necessary to determine whether the circuit we tentatively identified is specifically linked to OST. Ironically, the robust efficacy of OST may have mitigated our ability to discern relationships between COSS-P improvements and iFC changes at the individual level (data not shown). All participants in the OST group improved substantially, thus limiting the range of possible correlations between change in COSS-P scores and iFC. Finally, the generalizability of our findings to specific diagnoses (e.g., autism-spectrum disorder) should be approached with caution given the small number of such participants. Future research targeting specific diagnostic groups is necessary to clarify these associations.

In summary, we identified a possible substrate of improvement in organizational skills, but in the unexpected direction, and the results were not confirmed by two follow-up analyses. Given the rapid rate of development of analytical methods, we suspend judgment regarding the potential brain substrates of OST and provide full de-identified data for future explorations by the scientific community.

## Data Availability

The datasets presented in this study can be found in online repositories. The names of the repository/repositories and accession number(s) can be found below: the pipeline configuration file is available in https://github.com/luisagulleiro/OrganizationalSkillsTraining Participant-specific diagnoses and other phenotypic deidentified data are provided at: https://openneuro.org/datasets/ds005566.

## Footnotes

1
https://osf.io/5m5sx/

2
http://rfmri.org/DPARSF

3
http://www.fil.ion.ucl.ac.uk/spm

4
https://fcp-indi.github.io/

5
https://github.com/luisagulleiro/OrganizationalSkillsTraining

6
https://osf.io/5m5sx/

7
https://openneuro.org/datasets/ds005566

8
https://osf.io/5m5sx/

9
https://openneuro.org/datasets/ds005566
