# Supplementary material for: Brain plasticity underlying acquisition of new organizational skills in children: A Rashomon analysis
Source: Front Neuroimaging. 2025 Dec 9;4:1671310. doi: 10.3389/fnimg.2025.1671310 (PMC12723142; doi:10.3389/fnimg.2025.1671310)
Supplement: Supplementary file 1 [file Supplementary_file_1.docx]

**SUPPLEMENTARY METHODS**

**CONN toolbox**

Analyses of fMRI data were performed using CONN^1^ (RRID:SCR_009550) release 22.v2407^2^ and SPM^3^ (RRID:SCR_007037) release 12.7771. Functional and anatomical data were preprocessed using a modular preprocessing pipeline^4^ including realignment with correction of susceptibility distortion interactions, slice timing correction, outlier detection, direct segmentation and MNI-space normalization, and smoothing.

Functional data were realigned using SPM realign & unwarp procedure,^5^ where all scans were coregistered to a reference image (first scan of the first session) using a least squares approach and a 6 parameter (rigid body) transformation,^6^ and resampled using b-spline interpolation to correct for motion and magnetic susceptibility interactions. Temporal misalignment between different slices of the functional data was corrected following SPM slice-timing correction procedure,^7,8^ using sinc temporal interpolation to resample each slice BOLD timeseries to a common mid-acquisition time. Potential outlier scans were identified using ART^9^ as acquisitions with framewise displacement above 0.9 mm or global BOLD signal changes above 5 standard deviations,^10,11^ and a reference BOLD image was computed for each subject by averaging all scans excluding outliers. Functional and anatomical data were normalized into standard MNI space, segmented into grey matter, white matter, and CSF tissue classes, and resampled to 2 mm isotropic voxels following a direct normalization procedure^11,12^ using SPM unified segmentation and normalization algorithm^13,14^ with the default IXI-549 tissue probability map template. Last, functional data were smoothed using spatial convolution with a Gaussian kernel of 4 mm full width half maximum.

In addition, functional data were denoised using a standard denoising pipeline^15^ including the regression of potential confounding effects characterized by white matter timeseries (5 CompCor noise components), CSF timeseries (5 CompCor noise components), motion parameters and their first order derivatives (12 factors),^16^ outlier scans (below 23 factors),^10^ and linear trends (2 factors) within each functional run, followed by bandpass frequency filtering of the BOLD timeseries^17^ between 0.01 Hz and 0.1 Hz. CompCor^18,19^ noise components within white matter and CSF were estimated by computing the average BOLD signal as well as the largest principal components orthogonal to the BOLD average, motion parameters, and outlier scans within each subject's eroded segmentation masks. From the number of noise terms included in this denoising strategy, the effective degrees of freedom of the BOLD signal after denoising were estimated to range from 140.7 to 144.3 (average 143.4) across all subjects.^11^

**SUPPLEMENTARY RESULTS (WITHOUT GLOBAL SIGNAL REGRESSION)**

**Effect of OST on brain connectivity**

*Data Processing Assistant for Resting-State fMRI (DPARSF)*

After controlling for age and sex assigned at birth, linear regression models yielded a statistically significant effect of treatment group on changes in the intrinsic functional connectivity (post – pre; ΔiFC) between dACC and aVS-DMN (β = 0.122, 95% CI [0.011, 0.233], p = 0.031). The intervention explained 7.5% of the variance in ΔiFC between dACC and aVS-DMN (Cohen’s f² = 0.62). The immediate treatment group showed a statistically significant increase in iFC between dACC and aVS-DMN (mean [SD] ΔiFC = 0.089 [0.040], 95% CI [0.009, 0.168]), while the waitlist group showed a non-significant decrease in iFC between dACC and aVS-DMN (mean [SD] ΔiFC = -0.033 [0.039], 95% CI [-0.112, 0.045]). No statistically significant group differences were found in ΔiFC between dACC and aVS-FP (β = -0.079, 95% CI [-0.199, 0.041], p = 0.190) or between dACC and aVS-LIM (β = 0.005, 95% CI [-0.088, 0.097], p = 0.920).

*Configurable Pipeline for the Analysis of Connectomes (C-PAC)*

Findings from C-PAC yielded no statistically significant group differences in ΔiFC between dACC and the aVS ROIs: aVS-DMN (β = 0.05, 95% CI [-0.068, 0.161], p = 0.413), dACC and aVS-FP (β = -0.009, 95% CI [-0.126, 0.109], p = 0.882) and dACC and aVS-LIM (β = -0.053, 95% CI [-0.164, 0.056], p =0.329).

**REFERENCES**

1. Whitfield-Gabrieli S, Nieto-Castanon A. Conn: a functional connectivity toolbox for correlated and anticorrelated brain networks. *Brain Connect*. 2012;2(3):125-41. doi:10.1089/brain.2012.0073

2. Nieto-Castanon A, Whitfield-Gabrieli S. CONN functional connectivity toolbox: RRID SCR_009550, release 22. 2022;doi:doi:10.56441/hilbertpress.2246.5840

3. Penny WD, Friston KJ, Ashburner JT, Kiebel SJ, Nichols TE. *Statistical parametric mapping: the analysis of functional brain images*. Elsevier; 2011.

4. Nieto-Castanon A. FMRI minimal preprocessing pipeline. *Handbook of functional connectivity Magnetic Resonance Imaging methods in CONN*. Hilbert Press; 2020:3-16.

5. Andersson JL, Hutton C, Ashburner J, Turner R, Friston K. Modeling geometric deformations in EPI time series. *Neuroimage*. 2001;13(5):903-919.

6. Friston KJ, Ashburner J, Frith CD, Poline JB, Heather JD, Frackowiak RS. Spatial registration and normalization of images. *Human brain mapping*. 1995;3(3):165-189.

7. Henson R, Buechel C, Josephs O, Friston K. The slice-timing problem in event-related fMRI. *NeuroImage*. 1999;9:125-.

8. Sladky R, Friston KJ, Tröstl J, Cunnington R, Moser E, Windischberger C. Slice-timing effects and their correction in functional MRI. *Neuroimage*. 2011;58(2):588-594.

9. *Artifact detection tools (ART)*. Version Release Version, 7(19), 11. 2011.

10. Power JD, Mitra A, Laumann TO, Snyder AZ, Schlaggar BL, Petersen SE. Methods to detect, characterize, and remove motion artifact in resting state fMRI. *neuroimage*. 2014;84:320-341.

11. Nieto-Castanon A. Preparing fMRI data for statistical analysis. *arXiv preprint arXiv:221013564*. 2022;

12. Calhoun VD, Wager TD, Krishnan A, et al. *The impact of T1 versus EPI spatial normalization templates for fMRI data analyses*. 2017. 1065-9471.

13. Ashburner J, Friston KJ. Unified segmentation. *neuroimage*. 2005;26(3):839-851.

14. Ashburner J. A fast diffeomorphic image registration algorithm. *Neuroimage*. Oct 15 2007;38(1):95-113. doi:10.1016/j.neuroimage.2007.07.007

15. Nieto-Castanon A. FMRI denoising pipeline. *Handbook of functional connectivity Magnetic Resonance Imaging methods in CONN*. Hilbert Press; 2020:17-25.

16. Friston KJ, Williams S, Howard R, Frackowiak RS, Turner R. Movement‐related effects in fMRI time‐series. *Magnetic resonance in medicine*. 1996;35(3):346-355.

17. Hallquist MN, Hwang K, Luna B. The nuisance of nuisance regression: spectral misspecification in a common approach to resting-state fMRI preprocessing reintroduces noise and obscures functional connectivity. *Neuroimage*. 2013;82:208-225.

18. Behzadi Y, Restom K, Liau J, Liu TT. A component based noise correction method (CompCor) for BOLD and perfusion based fMRI. *Neuroimage*. 2007;37(1):90-101.

19. Chai XJ, Castañón AN, Öngür D, Whitfield-Gabrieli S. Anticorrelations in resting state networks without global signal regression. *Neuroimage*. 2012;59(2):1420-1428.
